# Carbohydrates digestion and metabolism in the spiny lobster (*Panulirus argus*): biochemical indication for limited carbohydrate utilization

**DOI:** 10.7717/peerj.3975

**Published:** 2017-11-03

**Authors:** Leandro Rodríguez-Viera, Erick Perera, Vivian Montero-Alejo, Rolando Perdomo-Morales, Tsai García-Galano, Gonzalo Martínez-Rodríguez, Juan M. Mancera

**Affiliations:** 1 Center for Marine Research, University of Havana, Havana, Havana, Cuba; 2 Faculty of Marine and Environmental Sciences, Campus de Excelencia Internacional del Mar (CEIMAR), University of Cadiz, Puerto Real, Cadiz, Spain; 3 Nutrigenomics and Fish Growth Endocrinology, Institute of Aquaculture Torre de la Sal, IATS-CSIC, Castellón, Valencia, Spain; 4 Department of Biochemistry, Center for Pharmaceuticals Research and Development, Havana, Cuba; 5 ICMAN-CSIC, Instituto de Ciencias Marinas de Andalucía, Puerto Real, Cadiz, Spian

**Keywords:** α-amylase, Carbohydrate, Energy metabolism, Crustaceans, Glycemia, Gene expression, *Panulirus argus*, Lobster, Carbohydrate digestion

## Abstract

As other spiny lobsters, *Panulirus argus* is supposed to use preferentially proteins and lipids in energy metabolism, while carbohydrates are well digested but poorly utilized. The aim of this study was to evaluate the effect of dietary carbohydrate level on digestion and metabolism in the spiny lobster *P. argus*. We used complementary methodologies such as post-feeding flux of nutrients and metabolites, as well as measurements of α-amylase expression and activity in the digestive tract. Lobsters readily digested and absorbed carbohydrates with a time-course that is dependent on their content in diet. Lobster showed higher levels of free glucose and stored glycogen in different tissues as the inclusion of wheat flour increased. Modifications in intermediary metabolism revealed a decrease in amino acids catabolism coupled with a higher use of free glucose as carbohydrates rise up to 20%. However, this effect seems to be limited by the metabolic capacity of lobsters to use more than 20% of carbohydrates in diets. Lobsters were not able to tightly regulate α-amylase expression according to dietary carbohydrate level but exhibited a marked difference in secretion of this enzyme into the gut. Results are discussed to highlight the limitations to increasing carbohydrate utilization by lobsters. Further growout trials are needed to link the presented metabolic profiles with phenotypic outcomes.

## Introduction

Despite significant achievements made on the larval propagation of spiny lobsters (Barnard, Johnston & Phillips, 2011; Perera & Simon, 2014), a major interest remains on the growout of lobsters based on the capture of wild seed (Williams, 2007; Perera & Simon, 2014; Radhakrishnan, 2015), especially for fast-growing tropical species (e.g., *Panulirus argus*, *Panulirus ornatus*) (Jeffs & David, 2003; Williams, 2007; Nguyen, Long & Hoc, 2009). However, the absence of appropriate diets is so far the main impediment to the sustainable expansion of this activity (Williams, 2007); during growout, spiny lobsters are currently fed with trash fish (Perera & Simon, 2014; Radhakrishnan, 2015), with downstream negative effects such as environmental pollution, poor feed conversion, appearance of emerging diseases, and overpressure on wild fish stocks (Perera & Simon, 2014; Radhakrishnan, 2015). Although the nutritional requirements of some spiny lobsters have been evaluated, growth rates with formulated diets are still low for most species (Crear et al., 2000; Glencross et al., 2001; Smith et al., 2003; Ward et al., 2003; Johnston et al., 2003; Smith, Williams & Irvin, 2005; Simon & Jeffs, 2008).

It is recognized that problems for feeding spiny lobsters with formulated diets are partially due to gaps in our knowledge on their digestive physiology and metabolism (Perera & Simon, 2014). Different studies in the spiny lobster *P. argus* digestive physiology have been focused on protein digestion (Perera et al., 2008a; Perera et al., 2008b; Perera et al., 2010a; Perera et al., 2010b; Perera et al., 2012a; Perera et al., 2012b), while digestion of other nutrients has received less attention. From a diet development perspective, carbohydrates (CHs) would provide a cheap source of energy, which is assumed to be advantageous in term of growth and profitability. However, there is no evidence of a significant use of CHs for energy in spiny lobsters, though some energy appears to be derived from glycogen of the digestive gland (DG) during short term fasting (Simon & Jeffs, 2013). *P. argus* is currently supposed to use preferentially proteins and lipids in energy metabolism (Perera et al., 2005), and studies in this and other spiny lobster species have provided indication that several CHs are well digested but poorly utilized (Simon, 2009a; Simon, 2009b; Simon, 2009c; Simon & Jeffs, 2013; Rodríguez-Viera et al., 2014). The metabolism of other crustaceans such as penaeid shrimps is also directed to the use of proteins and lipids for energy, but CHs can spare dietary proteins to a certain extent (Cuzon et al., 2000).

Previous studies indicated that native wheat flour (∼70% starch) results in a gradual digestion and liberation of glucose to the hemolymph and may have the major potential for optimizing energy metabolism of lobsters (Simon, 2009a; Simon, 2009b; Simon, 2009c; Simon & Jeffs, 2011; Simon & Jeffs, 2013; Rodríguez-Viera et al., 2014). Although different factors affecting starch hydrolysis come from the CH source itself (e.g., granule size and shape, amylose content), the true digestion rate arises from the interaction between CHs and digestive carbohydrases. As in other crustaceans (Pavasovic et al., 2004), adaptation of α-amylase to dietary CHs has been demonstrated in the spiny lobster *J. edwardsii*. The α-amylase activity in this species significantly decreased with increasing inclusion of CHs in diet (Simon & Jeffs, 2011; Simon & Jeffs, 2013). Preliminary gene expression analysis in *P. argus* suggested that dietary regulation of α-amylase activity in spiny lobsters may be exerted at the transcriptional level (Rodríguez-Viera et al., 2016). However, this flexibility in gene expression of *P. argus* α-amylase seems not sufficient to control CHs digestion whenlobsters are fed on formulated diets, probably due to low *K*_m_ of the enzyme (Rodríguez-Viera et al., 2016) and its high activity in the conditions of the gastric juice (Perera et al., 2008a; Perera et al., 2008b).

The aim of this study was to evaluate the effect of CH level on digestive α-amylase transcriptional regulation, CH digestion and metabolism in the spiny lobster *P. argus*. Complementary methodologies such as post-feeding fluxes of nutrients and metabolites, as well as assessments of activity and gene expression of α-amylase in the digestive tract were used. Results suggest that regulatory mechanisms of digestive α-amylases in the lobster are not well developed at the transcriptional level, with more complexity added at the level of secretion of the enzymes. Furthermore, this is probably the first study providing biochemical evidence of the protein sparing effect of dietary CHs in spiny lobsters. However, this effect seems to be limited by the metabolic capacity of lobster to use diet-derived glucose, with no improvement with increments in dietary CHs beyond 20%.

## Materials & Methods

### Experimental diets and feeding trial

Three experimental diets were formulated to contain different inclusion levels of CHs (6%, 20%, and 35%) (Table 1). All feedstuffs were obtained from commercial suppliers (Table 1). Pellets were made as described in a previous work (Perera et al., 2012a; Perera et al., 2012b). Spiny lobsters were collected in the Gulf of Batabanó, Cuba, under permission of the Fisheries Regulator Department from the Ministry of the Fishing Industry of Cuba. The feeding trial was conducted at the Center for Marine Research of the University of Havana, Cuba, in a facility equipped recirculated sea water, constant aeration, and photoperiod of 12 h light: 12 h darkness. Water quality was monitored twice a week: ∼26 °C, pH∼8.0, salinity 36 ups, oxygen ∼6.0 mg/L, and ammonia-N∼0.07 mg/L. Each experimental diet was sorted at random to six lobsters (∼250 g), housed individually in 60 L tanks. Only intermolt individuals (Drach & Tchemigovzteff, 1967; Lyle & MacDonald, 1983) were used.

**Table 1:** Formulation (%) and proximate composition of the experimental diets.

Ingredients	6%	20%	35%
Fish meal^a^	35	32	28.7
Squid meal^b^	15	15	15
Gelatin^c^	5	5	5
Wheat flour^d^	3	22.2	42.4
Fish oil^e^	1.9	1.9	1.9
Lecithin^f^	2	2	2
Cholesterol^g^	1	1	1
Vit & Min Premix^h^	1	1	1
Phosphate/carbonate^i^	2	2	2
Attractants^j^	1	1	1
Talc^d^	33.1	16.9	–
** Total**	100	100	100
Proximate composition^k^			
Crude protein	41.70	45.13	43.47
Crude lipid	9	9	10
Carbohydrate	5.75	19.82	36.45
Ash	43.65	27.05	9.08

**Notes.**

Pellets contained 10–12% of water.

a Protazul 65: 65% proteins, 12% lipids, 5% moisture.

b Imperial Baits Carptrack products: 70% proteins, 15% lipids, 6% moisture.

c G2500; Sigma-Aldrich, St. Louis, MO, USA.

d Commercially available regular feedstuff.

e Fisheries Research Center Laboratory, Havana, Cuba.

f Calbiochem (429415); Merck Chemicals Ltd., Billerica, MA, USA.

g Sigma-Aldrich (C8667).

h Vitamins and Minerals Premix from DIBAQ-Aquaculture, Segovia, Spain, containing (per kg of feed): vitamin A 15,000 IU, vitamin D3 3000 IU, vitamin E 180 mg, vitamin K 15 mg, vitamin B1 37.5 mg, vitamin B2 37.5 mg, vitamin B6 24.75 mg, vitamin B12 0.045 mg, vitamin H 1.14 mg, D-pantothenic acid 120 mg, nicotinic acid 225 mg, vitamin C 300 mg, folic acid 11.24 mg, Inositol 112.5 mg, zinc 75 mg, selenium 0.3 mg, magnesium 86.25 mg, copper 2.25 mg, manganese 22.5 mg, iodine 7.5 mg, iron 3 mg, cobalt 0.3 mg.

i Dicalcium phosphate/Calcium carbonate (1:2); Santa Cruz Fish Feed Factory, Camagüey, Cuba.

j Taurine (T0625; Sigma-Aldrich, St. Louis, MO, USA) 500 mg/kg diet, Glycine (G8898; Sigma-Aldrich, St. Louis, MO, USA) 500 mg/kg diet.

k Measured as described before in Rodríguez-Viera et al. (2014).

Lobsters were acclimatized for one week to experimental diets by gradually reducing fish flesh as food until they consumed only the pellets. The ration was progressively adjusted to 2% of body weight per day (BW day^−1^). This ration is sufficient for lobsters to feed close to satiation (Simon, 2009a; Simon, 2009b; Simon, 2009c). After this period, lobsters were fasted for two days and then they were provided with a 2% BW ration of the experimental diets for serial collection of gastric juice and hemolymph.

### Serial collection of gastric juice and hemolymph

Samples of gastric fluid were obtained through the oral cavity using insulin syringes with a plastic cannula over the needle as described before (Perera et al., 2012b). Gastric juice was not sampled before feeding as this affects feed intake. Serial samples (∼100 µL) of gastric juice were taken at 2, 6, 12, 24 and 30 h after ingestion, centrifuged at 10,000× g for 10 min, frozen in liquid nitrogen and stored at −80 °C. Samples were rapidly taken (less than 1 min) to avoid excessive stress. Hemolymph was not sampled prior to feeding, as this is known to affect feed intake in other spiny lobster species (Simon, 2009a) and by previous observation in our laboratory (Rodríguez-Viera et al., 2014). Hemolymph sampling began 2 h after feeding, with additional samples at 6, 12, 24, and 30 h. Serial sampling of hemolymph in lobsters has little effect on hemolymph glucose concentration (Radford et al., 2005; Rodríguez-Viera et al., 2014). Hemolymph samples (500 µL) were taken from the sinus of the 4th walking legs (Perdomo-Morales et al., 2007) in 1 mL pyrogen free syringes containing 500 µL of precooled anticoagulant solution (400 mM NaCl, 10 mM KCl, 10 mM HEPES, 20 mM EDTA, pH 7.3) (Hernandez-Lopez et al., 2003). An additional group of six lobsters were fed with fresh fish as control and sampled as above.

### Time-course of proteins and glucose in gastric juice and hemolymph

Soluble protein in gastric juice was measured as a sign of solubilization of dietary protein and enzyme secretion into the foregut. Gastric juice glucose was measured as indicator of the rate of CHs hydrolysis in the foregut. The glycemic prandial response was analyzedas indicator of digestibility and absorption of dietary CHs in lobsters (Rodríguez-Viera et al., 2014; Simon, 2009a; Radford et al., 2005) and protein in the hemolymph as a sign of their digestion and absorption. Soluble protein concentrations were quantified by the Lowry method using bovine serum albumin as standard (Lowry et al., 1951). Glucose level was determined using a HELFA^®^ RapiGluco-Test glucose oxidase kit (Quimefa Biological Products Inc., Havana, Cuba).

### Amylase activity

Amylase activity was measured as described before (Rodríguez-Viera et al., 2016) in a mixture composed of 5 µL of DG extract or gastric juice and 200 µL of assay buffer (50 mM MES (2-(N-morpholino) ethanesulfonic acid), pH 5.5), with 0.5 mM 2-Chloro-4-nitrophenyl-a-D-maltotrioside (CNP-G3) as the substrate. CNP released was measured (at 405 nm and 37 °C) kinetically for 10 min in an ELx808IU microplate reader. Initial velocities were obtained using the software KC4 version 3.4 (BioTek Instruments, Winooski, VT, USA). The extinction coefficient of p-nitrophenol at 405 nm (reaction volume of 205 µL) was 9.774 mM^−1^ cm^−1^. A unit of amylase activity was defined as the amount of enzyme that produces 1 μmol p-nitrophenol/minute. Amylase activity was expressed per volume of gastric juice (µL) or DG weight (mg).

### Metabolites in digestive gland and muscle

After the 30 h time-course sampling of gastric juice and hemolymph, lobsters were fed for one month with the corresponding diets, left unfed for 48 h, and then fed again with the respective diets (Rodríguez-Viera et al., 2014). They were killed 24 h later in ice-cold water to remove DG, muscle, and hemolymph samples, which were immediately frozen in liquid nitrogen and freeze-dried for metabolite and metabolic enzyme measurements. Samples of ∼20 mg were homogenized in 1 mL water, centrifuged (30 min, 10,000× g, 4 °C), and the supernatant taken to assess tissue metabolites. Before centrifugation, an aliquot was taken for triglyceride (TG) determination. Soluble protein and glucose concentrations were measured as detailed above. Free amino acid concentration was assessed colorimetrically by the nynhidrin method (Yemm, Cocking & Ricketts, 1955; Rosen, 1957) with L-alanine as the standard. TG and lactate concentrations were measured using the commercial kits TAG (Spinreact, Girona, Spain) and Lactate (Spinreact, Girona, Spain), respectively. Glycogen concentration was assessed by the breakdown of glycogen by amyloglucosidase (Keppler & Decker, 1974) and the determination of resultant glucose by a commercial kit (Spinreact, Girona, Spain) as in our previous work (Rodríguez-Viera et al., 2014).

### Metabolic enzymes in HP and muscle

The activities of enzymes from different metabolic pathways were quantified in two key tissues for lobster metabolism, DG and muscle. Lyophilized samples of DG and muscle were homogenized in 10 volumes of ice-cold buffer (50 mM imidazole hydrochloride, pH 7.5, 1 mM 2-mercaptoethanol, 50 mM sodium fluoride, 4 mM EDTA, 250 mM sucrose, and 0.5 mM PMSF). Homogenates were centrifuged for 30 min at 10,000× g and supernatants used for assays. Enzymes activities measured were: hexokinase (HK, EC 2.7.1.11), glycerol-3-phosphate dehydrogenase (G3PDH, EC 1.1.1.8), pyruvate kinase (PK, EC 2.7.1.40), L-lactate dehydrogenase (LDH, EC 1.1.1.27), fructose 1,6-biphosphatase (FBPase, EC 3.1.3.11), glycogen phosphorylase (GPase, EC 2.4.1.1), glucose-6-phosphate dehydrogenase (G6PDH, EC 1.1.1.49), aspartate transaminase (AST, EC 2.6.1.1), alanine transaminase (ALT, EC 2.6.1.2), glutamate dehydrogenase (GDH, EC 1.4.1.2), and 3-hydroxyacyl-CoA dehydrogenase (HOAD, EC 1.1.1.35). The amount of sample was set to ensure initial velocities. Conditions for enzyme assays (e.g., buffer composition, cofactors, additional enzymes for coupled enzyme assays, and electron donor (NADH) or acceptors (NADP, NAD)) were according to Laiz-Carrión et al. (2003) and Sangiao-Alvarellos et al. (2003). Substrates were as in our previous work (Rodríguez-Viera et al., 2014): 5 mM D-glucose for HK and PK, 0.2 mM dihydroxyacetone phosphate for G3PDH, 6.25 mM lactic acid for LDH, 0.1 mM fructose-1,6-bisphosphate for FBPase, 5 mg/mL glycogen for GPase, 1 mM glucose-6-phosphate for G6PDH, 10 mM L-aspartate for AST, 7.5 mM L-alanine for ALT, 1.40 mM α-ketoglutarate for GDH, and 0.1 mM acetoacetyl-CoA for HOAD. Reactions without substrates were performed as negative controls. Reaction rates of enzymes were determined in duplicate by the increase or decrease in absorbance at 340 nm and 37 °C, as a result of NADPH production for HK, LDH, FBPase, GPase, and G6PDH activities, or NADH consumption for G3PDH, PK, AST, ALT, GDH, HOAD activities, respectively. All assays were performed using a Bio-Tek PowerWave 340 Microplate spectrophotometer using KCjunior Data Analysis Software (Bio-Tek Instruments, Winooski, VT, USA). One unit of enzyme activity (U) was defined as the amount of enzyme needed to transform 1 µmoL of substrate or produce 1 µmoL of product per min. Enzyme activity was expressed as U/mg of soluble protein.

### Effects of carbohydrate level on lobster *α*-amylase gene expression and activity

Amylase gene expression and activity were assessed in lobsters (*n* = 6 per diet) acclimated to the three experimental diets and fresh fish for one month, left unfed for 48 h, then fed again with the respective diets, and then killed 24 h after last ingestion. For amylase activity determination, DGs were homogenized with chilled Milli-Q^®^ water (90 mg/500 µL) using a glass piston homogenizer and the homogenates were centrifuged at 10,000× g, 30 min at 4 °C. The resultant upper lipid layers were discarded and the remaining supernatants stored at −80 °C. Samples for gene expression analyses were immediately placed in RNA*later* at 4 °C for 24 h and then stored at −20 °C until total RNA extraction.

Total RNA was isolated from individual DGs using an Ultra-Turrax^®^ T25 (IKA^®^-Werke) and the illustra™ RNAspin Mini Kit (GE Healthcare, Dornstadt, Germany). Concentration of total RNA was measured at 260 nm with the BioPhotometer Plus (Eppendorf), and its quality was determined in an Agilent 2100 Bioanalyzer (Agilent Technologies, Santa Clara, CA, USA) using the Agilent RNA 6000 Nano Kit. Specific primers (Table 2) were designed using the software Primer3 v.0.4.0 (http://frodo.wi.mit.edu/) for assessing the relative expression of α-amylase and elongation factor 1 alpha (*ef1a*) as the internal reference gene (Perera et al., 2010a; Perera et al., 2010b). *ef1a* showed low variability (less than 0.20 Ct) among experimental groups. Primers were synthesized by IDT (Integrated DNA Technologies, Leuven, Belgium). First, 500 ng of total RNA were reverse-transcribed in a 20 µL reaction using the qScript™cDNA synthesis kit (Quanta BioSciences) for 5 min at 22 °C, 30 min at 42 °C, and 5 min at 85 °C. qPCR conditions were optimized (Rodríguez-Viera et al., 2016), and different amounts of cDNA were used in triplicate (6 points of serial 1/5 dilutions from 10 ng to 3.2 pg per reaction) as templates to check the assay linearity (*R*^2^) and amplification efficiency (E) (Rodríguez-Viera et al., 2016). Assay was linear along all six serial dilutions (*R*^2^ = 0.999, *E* = 98.6), thus 10 ng of cDNA per reaction were further used in qPCR reactions; qPCR was performed with CFX Connect™ Real-Time System (BIO-RAD, Madrid, Spain). Each 10 µL reaction mixture contained 0.5 µL at 400 nM of each specific forward and reverse primer, and 5 µL of PerfeCTa SYBR^®^ Green FastMix™ (Quanta Biosciences, Gaithersburg, MD, USA) in Hard-Shell^®^ PCR Plates, 96 wells, thin-wall, covered with Microseal^®^ ‘B’ seal film (BIO-RAD). Control reactions with RNAse-free water (NTC) and RNA instead of cDNA (NRT) were included to ensure the absence of contamination or genomic DNA. qPCR thermal profile was: 95 °C, 10 min; (95 °C, 20 s; 60 °C, 35 s) X 40 cycles; melting curve (60 °C to 95 °C, 0.5 °C/5 s)) (Rodríguez-Viera et al., 2016). Relative quantification was performed using the 2^−ΔΔCT^ method (Livak & Schmittgen, 2001) corrected for efficiency of the standard curve (Pfaff, 2001).

**Table 2:** Primers used in this study to quantify the relative expression (qPCR) of α-amylase from *P. argus*.

qPCR primers	Nucleotide sequence	Amplified size
EF1- α Fw	5′-CCAGTAGACAAACCACTTCG-3′	532–551
EF1- α Rv	5′-CATACCTGGCTTCAAGATGC-3′	620–639
Pa-qPCR-AMY-Fw	5′-GAGTGACGGAGTTCAAGTACGG-3′	841–862
Pa-qPCR-AMY-Rv	5′-GTCGTGGTTGTCGATGAAGAC-3′	980–1,000

### Statistical analyses

Only results from lobsters in intermolt stage C were analyzed as molt stage has been found to affect digestive enzyme activities in *P. argus* (Perera et al., 2008b). All data were checked for normality and homogeneity of variance using Kolmogorov–Smirnov and Levene’s tests, respectively, with *P* ≤ 0.05. Metabolic enzymes and metabolites in digestive gland, hemolymph, and muscle 24 h after ingestion were analyzed by one-way ANOVA (*P* ≤ 0.05). Data from the time-course in gastric juice and hemolymph after ingestion were subjected repeated measures ANOVA (*P* ≤ 0.05), with sampling time as the within subject (repeated measure) factor, and diet as the between group factor. Additionally, one-way ANOVA analyses (*P* ≤ 0.05) were performed for each time to compare the single main effect diet. The Tukey’s test (*P* ≤ 0.05) was used to determine differences among means. The software package Statistica 7.0 (StatSoft Inc., Tulsa, OK, USA) was used for all tests and figures were produced by GraphPad Prism 5.00 (GraphPad Software, Inc., San Diego, California, US) (Rodríguez-Viera et al., 2016).

## Results

### Soluble proteins and amylase activity in gastric juice

**Figure 1:**
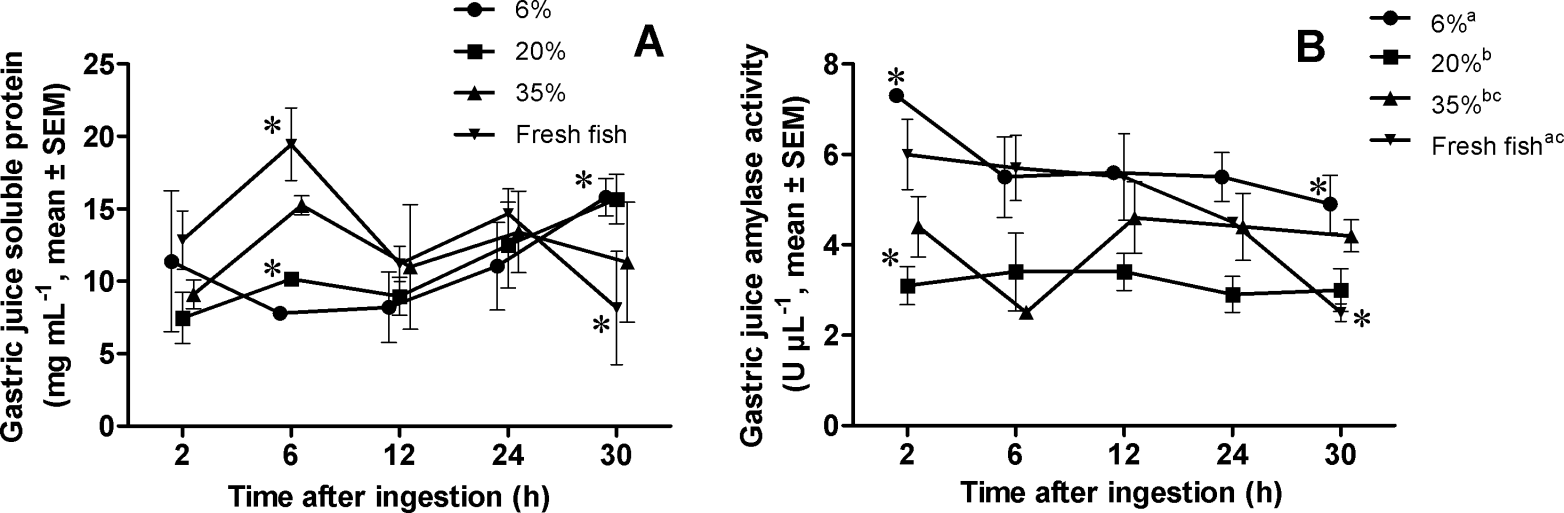
Soluble protein (A) and *α*-amylase activity (B) in the gastric juice of *Panulirus argus* after feeding. Diets were named according to the level of CH they contained (6%, 20%, 35%), and a control with fresh fish muscle. Each value is the mean ± SEM (*N* = 6 lobsters per diet). Differences among diets throughout the 30 h studied are marked by different superscript letters in legend (*P* ≤ 0.05). For each sampling time, statistically different (Tukey’s test, *P* ≤ 0.05) dietary treatments are indicated by asterisks.

Soluble proteins in the gastric juice did not vary among diets (Repeated measures ANOVA, *F* = 0.83, *P* > 0.05). Time and time x diet interaction were not significant factors (Repeated measures ANOVA, *F* = 1.39, *P* > 0.05) either. However, two apparent peaks of soluble proteins were found in the gastric juice at 6 and 24 h after ingestion, except with the diet containing 6% CH (Fig. 1A). Differences among diets (one-way ANOVA, *F* = 8.52, *P* ≤ 0.05) in soluble protein concentration of the gastric juice 6 h after ingestion were observed, being significantly higher in the diets with 35% CH and with fresh fish (Tuckey’s test, *P* ≤ 0.05) (Fig. 1A). Thirty hours after ingestion, basal values for soluble protein were only achieved by lobster fed fresh fish (one-way ANOVA, *F* = 5.10, *P* ≤ 0.05) (Fig. 1A). Lobsters from all treatments showed similar values at 12 and 24 h (Fig. 1A). Amylase activity per volume of gastric juice significantly varied among diets (Repeated measures ANOVA, *F* = 5.61, *P* ≤ 0.05), and through time (Repeated measures ANOVA, *F* = 3.21, *P* ≤ 0.05). Lobsters ingesting low CH diets (i.e., 6% CH diet and fresh fish) exhibited higher amylase activity in the gastric juice, especially during the first hours post-ingestion. After 30 h, only lobster fed the fresh fish decreased amylase activity in the gastric juice (Fig. 1B). Accordingly, the interaction time x diet resulted significant (Repeated measures ANOVA, *F* = 2.32, *P* ≤ 0.05). Amylase activity was higher for the 6% CH diet 2 h after ingestion (Tuckey’s test, *P* ≤ 0.05) (Fig. 1B).

### Time-course of glucose in gastric juice and hemolymph after feeding

**Figure 2:**
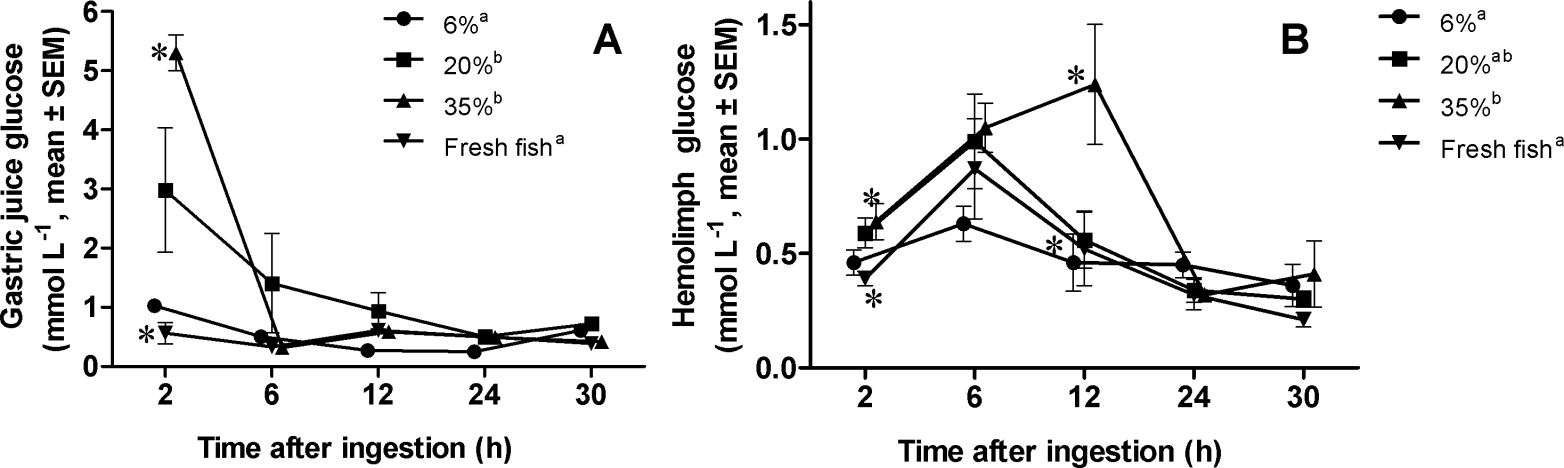
Glucose in gastric juice (A) and hemolymph (B), of *Panulirus argus* after feeding. Diets were named according to the level of CH they contained (6%, 20%, 35%), and a control with fresh fish muscle. Each value is the mean ± SEM (*N* = 6 lobsters per diet). Differences among diets throughout the 30 h studied are marked by different superscript letters in legend (*P* ≤ 0.05). For each sampling time, statistically different (Tukey’s test, *P* ≤ 0.05) dietary treatments are indicated by asterisks.

Free glucose concentration in gastric juice was affected by diets (Repeated measures ANOVA, *F* = 4.91, *P* ≤ 0.05) but no interaction time x diet was found (Repeated measures ANOVA, *F* = 1.96, *P* > 0.05). The main effect time had the major impact on the liberation of glucose into the gastric juice (Repeated measures ANOVA, *F* = 6.77, *P* ≤ 0.001), with a peak 2 h after ingestion (Fig. 2A). As expected, diets with 20 and 30% CH produced higher glucose levels than the 6% CH diet and fresh fish (Tuckey’s test, *P* ≤ 0.05) (Fig. 2A). However, 6 h after ingestion, free glucose levels in gastric juice did not differ among treatments. On the other hand, there were no differences in hemolymph free glucose levels due to the single main effect diet (Repeated measures ANOVA, *F* = 2.91, *P* > 0.05) (Fig. 2B). However, significant variation were found through time (Repeated measures ANOVA, *F* = 15.08, *P* ≤ 0.001) and a significant interaction time x diet was found (Repeated measures ANOVA, *F* = 2.03, *P* ≤ 0.001). Concentration of glucose in hemolymph increase with maximal values at 6 h (for 6% and 20% CH diets and fresh fish) or 12 h (for 35% CH diet) after ingestion (Fig. 2B). The concentration of glucose in the hemolymph 12 h after ingestion was significantly higher (one-way ANOVA, *F* = 3.41, *P* ≤ 0.05; Tuckey’s test, *P* ≤ 0.05) in 35% CH fed lobsters than in lobsters that ingested the other diets or fresh fish.

### Metabolites and metabolic enzymes in lobster tissues

Twenty-four hours after the ingestion of experimental diets and fresh fish the concentration of glucose (one way ANOVAs, *F* = 0.69, *P* > 0.05), lactate (*F* = 2.51, *P* > 0.05), and amino acids (*F* = 0.42, *P* > 0.05) in muscle did not vary among dietary treatments, and differences were found in TG (*F* = 16.54, *P* ≤ 0.05) and glycogen contents (*F* = 3.76, *P* ≤ 0.05). Higher glycogen content in muscle was found for the wheat diet at 35% (Table 3). In DG, differences were found among dietary treatments in content of glucose (*F* = 8.88, *P* ≤ 0.05), glycogen (*F* = 3.90, *P* ≤ 0.05), lactate (*F* = 3.51, *P* ≤ 0.05), amino acid (*F* = 3.22, *P* ≤ 0.05), but not in TG content (*F* = 0.28, *P* > 0.05) (Table 3). The highest difference was observed between 6% and 35% CH diets, while the 20% CH diet showed intermediate values (Table 3). At this sampling time, 24 h after ingestion, no significant differences were found in the concentration of glucose (*F* = 0.73, *P* > 0.05), lactate (*F* = 0.41, *P* > 0.05), and TG (*F* = 0.29, *P* > 0.05) in the hemolymph (Table 3). Differences were found in amino acid content in the hemolymph (*F* = 3.97, *P* ≤ 0.05), with major differences observed between lobsters ingesting fresh fish and the formulated diets (Tuckey’s test, *P* ≤ 0.05) (Table 3).

**Table 3:** Metabolite levels in digestive gland, muscle and hemolymph of the spiny lobster *Panulirus argus* 24 h after feeding. Diets were named according to the level of CH they contained (6%, 20%, 35%) and fresh fish muscle. All data are expressed on a dry matter basis. Each value is the mean ± SEM (*N* = 6 lobsters per diet). Different letters in the same row indicate significant differences among groups (one-way ANOVA, Tukey test, *P* ≤ 0.05).

Metabolites	Fresh fish	6%	20%	35%
	** Digestive gland**			
Glucose (mg g^−1^)	3.54 ± 0.63^a^	2.19 ± 0.69^a^	3.90 ± 0.48^a,b^	6.54 ± 0.59^b^
Glycogen (mg g^−1^)	0.36 ± 0.06^ab^	0.17 ± 0.06^a^	0.47 ± 0.11^ab^	0.51 ± 0.08^b^
Lactate (mg g^−1^)	0.54 ± 0.12^ab^	0.70 ± 0.16^ab^	0.69 ± 0.05^a^	0.74 ± 0.17^b^
Amino acid (mg g^−1^)	8.56 ± 1.09^ab^	9.97 ± 1.45^a^	9.46 ± 1.10^ab^	5.155 ± 0.74^b^
Triglyceride (mg g^−1^)	73.70 ± 9.53	55.27 ± 9.86	48.94 ± 12.59	41.58 ± 10.13
	**Muscle**			
Glucose (mg g^−1^)	18.48 ± 4.84	18.67 ± 4.23	12.03 ± 2.29	12.30 ± 5.47
Glycogen (mg g^−1^)	1.22 ± 0.18^b^	0.60 ± 0.09^b^	0.85 ± 0.33^b^	4.12 ± 1.04^a^
Lactate (mg g^−1^)	5.18 ± 0.89	5.04 ± 0.90	2.94 ± 0.38	3.18 ± 0.71
Amino acid (mg g^−1^)	23.04 ± 4.72	27.33 ± 5.27	20.39 ± 21.24	24.92 ± 5.65
Triglyceride (mg g^−1^)	3.14 ± 0.31^b^	10.14 ± 1.30^a^	4.25 ± 0.61^b^	4.88 ± 0.42^b^
	**Hemolymph**			
Glucose (mmol L^−1^)	1.22 ± 0.13	1.25 ± 0.12	1.06 ± 0.05	1.15 ± 0.12
Lactate (mg dL^−1^)	4.10 ± 0.53	3.73 ± 1.13	3.58 ± 0.55	3.95 ± 0.39
Amino acid (mmol dL^−1^)	0.51 ± 0.05^b^	1.03 ± 0.14^a^	1.18 ± 0.26^a^	0.89 ± 0.18^ab^
Triglyceride (mg dL^−1^)	6.21 ± 0.56	10.48 ± 2.54	5.73 ± 0.85	6.47 ± 0.96

Metabolic enzyme activities with differences among dietary treatments 24 h after feeding were (Table 4): HK (one way ANOVAs, *F* = 3.77, *P* ≤ 0.05), G3PDH (*F* = 14.70, *P* ≤ 0.05), PK (*F* = 13.24, *P* ≤ 0.05), FBP (*F* = 5.555, *P* ≤ 0.05), GPase (*F* = 6.429, *P* ≤ 0.05), and AST (*F* = 5.408, *P* ≤ 0.05) in muscle, and HK (*F* = 14.09, *P* ≤ 0.05), G3PDH (*F* = 14.70, *P* ≤ 0.05), FBP (*F* = 3.696, *P* ≤ 0.05), G6PDH (*F* = 7.438, *P* ≤ 0.0001), AST (*F* = 6.295, *P* ≤ 0.05), and GDH (*F* = 10.69, *P* ≤ 0.05) in the DG. No differences were found in the following enzyme activities: LDH (*F* = 1.151, *P* > 0.05), G6PDH (*F* = 0.239, *P* > 0.05), ALT (*F* = 2.699, *P* > 0.05), GDH (*F* = 0.130, *P* > 0.05), HOAD (*F* = 0.425, *P* > 0.05) in muscle, and PK (*F* = 0.914, *P* > 0.05), LDH (*F* = 1.419, *P* > 0.05), GPase (*F* = 1.652, *P* > 0.05), ALT (*F* = 1.807, *P* > 0.05) and HOAD (*F* = 0.216, *P* > 0.05) in the DG.

**Table 4:** Activity of key enzymes of intermediary metabolism in digestive gland and muscle of the spiny lobster *Panulirus argus* 24 h after feeding. Diets were named according to the level of CH they contained (6%, 20%, 35%), and fresh fish muscle. All enzyme activities are expressed as U mg protein^−1^. Each value is the mean ± SEM (*N* = 6 lobsters per diet). For each tissue, different letters in the same row indicate significant differences among groups (one-way ANOVA, Tukey test, *P* ≤ 0.05).

Route/Enzyme	Digestive gland				Muscle			
	Fresh fish	6%	20%	35%	Fresh fish	6%	20%	35%
Glycolysis								
HK	5.76 ± 0.83^b^	9,03 ± 0.92^b^	15.03 ± 1.16^a^	12.60 ± 0.75^a^	1.22 ± 0.30^b^	1,96 ± 0.65^ab^	1.26 ± 0.25^b^	2.48 ± 0.21^a^
G3PDH	1.00 ± 0.34^b^	2.24 ± 0.78^ab^	1.79 ± 0.70^ab^	4.15 ± 0.74^a^	12.13 ± 0.88^a^	4.68 ± 1.45^b^	2.15 ± 1.00^b^	3.39 ± 1.06^b^
PK	8.87 ± 1.82	8.04 ± 2.77	13.13 ± 2.94	14.67 ± 4.58	48.38 ± 7.11^a^	21.42 ± 3.44^b^	29.19 ± 3.43^b^	55.75 ± 4.22^a^
Gluconeogenesis								
LDH	170.2 ± 34.37	194.25 ± 94.05	173.28 ± 88.59	96.72 ± 30.11	12.14 ± 1.47	7.02 ± 3.80	13.61 ± 2.92	6.18 ± 0.48
FBPase	4.41 ± 1.19^a^	1.78 ± 0.30^ab^	1.97 ± 0.30^ab^	1.40 ± 0.77^b^	6.82 ± 2.18^ab^	2.98 ± 1.74^b^	4.03 ± 1.55^ab^	6.82 ± 0.94^a^
Glycogenolysis								
GPase	6.31 ± 1.41	9.43 ± 2.34	9.19 ± 1.95	4.02 ± 0.58	95.26 ± 8.57^b^	181.6 ± 24.67^ab^	225.7 ± 51.84^a^	100.3 ± 8.64^b^
Pentose shunt								
G6PDH	13.64 ± 1.84^a^	2.51 ± 0.30^b^	5.91 ± 1.02^b^	4.98 ± 1.64^b^	1.08 ± 0.33	1.16 ± 0.29	1.14 ± 0.13	1.43 ± 0.38
Amino acid								
AST	547.5 ± 82.68^a^	479.1 ± 98.06^a^	434.0 ± 49.56^a^	198.9 ± 37.96^b^	37.85 ± 4.30^ab^	53.28 ± 8.49^a^	25.69 ± 3.65^b^	34.88 ± 2.82^b^
ALT	3.53 ± 1.22	2.10 ± 0.57	2.31 ± 0.71	4.22 ± 0.41	6.0 ± 1.78	3.98 ± 0.64	3.14 ± 0.44	5.26 ± 1.20
GDH	287.9 ± 17.29^a^	166.0 ± 21.14^b^	167.5 ± 21.14^b^	127.1 ± 21.66^b^	32.53 ± 2.49	30.16 ± 7.20	28.03 ± 4.06	28.99 ± 4.38
Fatty acid								
HOAD	227.66 ± 20.70	221.26 ± 35.17	164.93 ± 32.79	228.29 ± 29.23	7.04 ± 1.16	6.84 ± 1.63	5.89 ± 0.45	5.41 ± 1.19

### Lobster *α*-amylase expression and secretion

**Figure 3:**
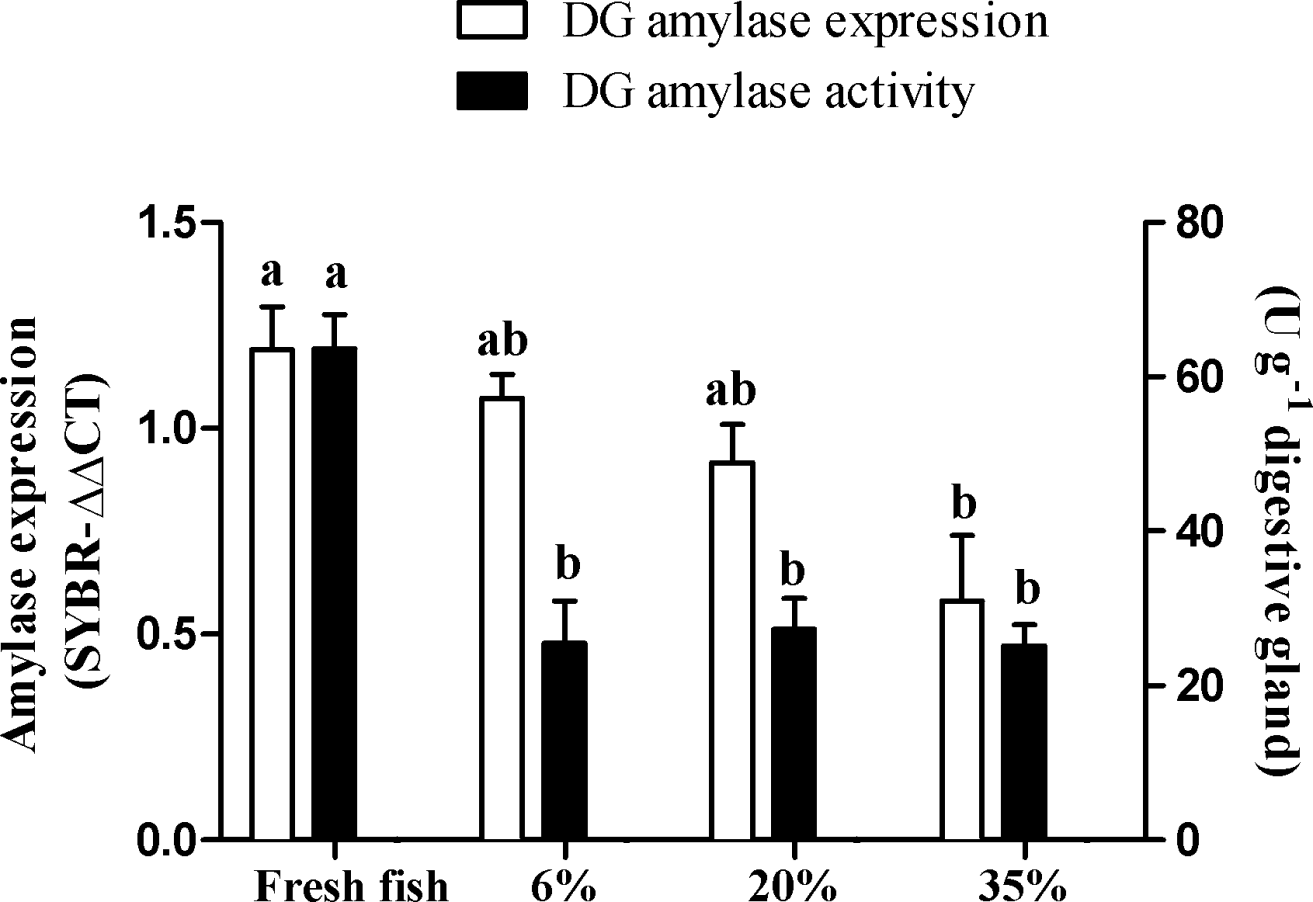
Alpha-amylase activity and gene expression in the digestive gland (DG) of *Panulirus argus* feeding. Diets were named according to the level of CH they contained (6%, 20%, 35%), and a control with fresh fish muscle. Values are means ± SEM (*N* = 6 lobsters per diet). Different letters above the bars indicate statistical differences according to the Tukey’s test (*P* < 0.05). Amylase activity in the DG was highly impacted by formulated diets feeding.

We have previously found that the expression of α-amylase in lobster may be regulated by the type of food ingested (Rodríguez-Viera et al., 2014). Thus, we next sought to establish whether different levels of dietary CHs are able to exert this transcriptional regulation. We fed lobster with fresh fish or the three formulated diets with 6%, 20%, and 35% CH (Table 1) and measured the expression and activity of α-amylase 24 h later. Twenty-four hours after ingestion, there were differences among diets in the expression level of α-amylase (one-way ANOVA, *F* = 3.892, *P* < 0.05, Tukey’s test, *P* < 0.05) (Fig. 3). Difference in α-amylase gene expression was only found between animals fed with fresh fish and the 35% CH diet. Lobsters fed with fresh fish had the highest α-amylase activity in the digestive gland (one-way ANOVA, *F* = 13.97, *P* < 0.0001, Tukey’s test, *P* ≤ 0.05), while no differences in activity were found in animals fed with the three formulated diets despite highly different CH content (Fig. 3).

## Discussion

Wheat flour is a good source of CHs for formulating diets for crustaceans such as the shrimp *L. vannamei* (Cousin et al., 1996), the European lobster *Homarus gammarus* (Glass & Stark, 1995) or the spiny lobsters *Jasus edwardsii* (Simon, 2009a) and *P. argus* (Rodríguez-Viera et al., 2014; Rodríguez-Viera et al., 2016); wheat flour apparent digestibility for *P. argus* is 91% (Rodríguez-Viera et al., 2014). In this study, it was assessed whether lobster digestion and metabolism may be optimized by managing the wheat content of diets. A previous study revealed that lobster feeding on the same level of CHs, but from different origins, exhibited similar levels of α-amylase activity in the gastric juice (Rodríguez-Viera et al., 2014). In this study, differences were observed in α-amylase activity in gastric juice between diets with different CHs content. In general, activity was significantly higher in the diet at 6% CH and with fresh fish, whereas the diets at 20 and 35% CH had lower values (Fig. 1B). This result suggests an adaptation to the usual diet composition. Similar results were found in the spiny lobster *J. edwwarsii*; in this other lobster species, lower values of α-amylase activity in the foregut and digestive gland were observed in individuals ingesting a formulated diet containing 36% wheat starch compared to those ingesting fresh food (Simon, 2009c). Differences in α-amylase activity induced by diets with different CHs inclusion have also been found in other crustaceans (Ding et al., 2017). Taken together with previous results and those obtained in this work, it is demonstrated that spiny lobsters are relatively insensitive to CHs source in terms of α-amylase secretion to the gastric juice, but that they modify the amount of α-amylase secreted according to the amount of starch in the usual diet.

As expected, free glucose values in gastric juice varied accordingly with the level of CH inclusion in diets, with higher values found in lobsters that ingested the 35% CH diet (Fig. 2A). After 6 h, free glucose levels in the gastric juice dropped for all treatments (Fig. 2A), indicating a rapid digestion and absorption of CHs. This agrees with a non-significant increase in soluble protein concentration at the gastric juice 6 h after ingestion (Fig. 1A), resembling previous observations in this and other spiny lobsters (Rodríguez-Viera et al., 2014; Simon, 2009c), and assumed to be due to dissolution of the feed and the increased enzyme secretion, including amylase (Perera et al., 2012a; Simon, 2009c). Accordingly, free glucose was observed to peak at 6 h in the hemolymph (Fig. 2B). This time course slightly differs from that observed in a previous study, in which glucose peaked 6 h after ingestion in the gastric juice (Rodríguez-Viera et al., 2014). Gastric juice glucose levels are impacted among other factors by water ingestion, secretion of gastric juice, and sampling artifacts, but in general, results show that starch in formulated diets are highly hydrolyzed and absorbed within the first 6 h after ingestion (Rodríguez-Viera et al., 2014; this work), with maximal hyperglycemic responses from 6 to 12 h after meal (Fig. 2B). Glycemia decreased in all treatments after 12 h until basal levels 24 h later. Nevertheless, hemolymph glucose started to decline in lobsters that ingested the diets with 35% CHs well after that in the other treatments, indicating slower metabolic use.

The capacity of spiny lobsters to use CHs as a source of energy has been a debated issue in recent years (Perera & Simon, 2014). Our results showed that lobsters fed regularly with 20% and 35% CH diets, HK activity is enhanced in the digestive gland, as well as PK activity in the muscle (Table 4). In general, the values of these two enzymes indicated that there was a stimulation of glycolysis with high CH diets. HK is one of the key enzymes in glycolysis, phosphorylating the glucose, and it is known to be indication of the preferential use of free glucose (Hochachka et al., 1971; Gaxiola et al., 2005); PK catalyzes the formation of pyruvate and is other key enzyme in glycolysis. Thus, it is plausible to postulate that by increasing dietary CHs in lobster diets, the glycolytic (and glycogen synthesis) use of CHs is stimulated. This seems to be not a direct postprandial response but evidence of an overall adjustment of intermediary metabolism, as measurements were taken in fasted lobsters. However, this is only possible up to a certain CHs level as no differences were found between 20 and 35% CH inclusion. According to our results, the phosphorylation capacity (HK activity) of the DG increased from 6% to 20% CH, but no further improved with 35% CH. Thus, the lobster DG has the same capacity to incorporate glucose to glycolysis or glycogen synthesis (no difference in glycogen content, Table 3) with these two diets. From a practical point of view, this evidences a bottleneck on the metabolic use of dietary CHs. Interestingly, HK activity increased in muscle as dietary CHs increased from 20% to 35%, suggesting that conversely to the DG, the muscle increased the use of free glucose under high CHs load both in energy metabolism (increased PK activity) and glycogen storage (more glycogen, Table 3). Surprisingly, higher glycogen content was found in the DG of lobsters fed the fresh fish, compared to the 6% CH diet (Table 3), in spite of fish having lower CH content (∼2%). This may be partially explained by higher FBPase activity, a key enzyme in gluconeogenesis, which may give lobsters the capacity to synthesize glucose from non-CH substrates (Tables 3 and 4). While previous studies have indicated that spiny lobsters have a limited capacity for glycogen synthesis during the feeding period in molt stage C (Travis, 1955; Simon & Jeffs, 2011; Rodríguez-Viera et al., 2014), our results point to a general effect of fresh fish meat on glycogen accumulation.

On the other hand, enzymes activities involved in amino acids catabolism such as AST activity tend to decrease both in the muscle and in the DG as dietary CHs increased (Table 4). In addition, GH activity, which is involved in ammonia formation (Mayzaud & Conover, 1988; Bidigare & King, 1981), mostly in the DG, was lower in lobsters fed on test diets, especially those with high CHs content (Table 4). Altogether, these biochemical results indicated that there is a protein sparing effect of dietary CHs as their inclusion level increased. This is probably the first study providing biochemical evidence, though indirect (i.e., not direct measurements of N-retention), of a protein sparing effect of CHs in spiny lobsters. However, this effect seems to be limited by the metabolic capacity of lobster to use diet-derived glucose, with no improvement with increments in CHs inclusion beyond 20%. On the other hand, while a previous study revealed that the use of wheat flour in diets improves *P. argus* utilization of fatty acids (FA) (Rodríguez-Viera et al., 2014), the present work shows that further improvement in FA utilization cannot be achieved by managing wheat flour inclusion. This suggestion derived from the absence of differences in HOAD activity (a key enzyme for FA β-oxidation) between the different diets or fresh fish meat (Table 4). However, lobster ingesting fish meat accumulated more fat in the DG, thus metabolic factors affecting dietary lipid storage or oxidation in lobsters deserve further investigation. It is apparent that an interaction occurs between carbohydrate content of diet and lipid utilization in *P. argus*. A previous study on the spiny lobster *J. edwardsii* showed that lobsters fed low CH, high lipid diets, were in the best nutritional condition, with higher lipid accumulation (Johnston et al., 2003). Interestingly, G6PDH, a key enzyme in the pentose shunt pathway (HMS), was significantly higher in the DG of lobsters that usually fed on fish meat than in lobsters fed the test diets (Table 4). Given that the HMS pathway provide NADPH for lipid biosynthesis, higher activity of G6PDH in DG of lobster feeding fresh fish would indicate a higher potential for lipid synthesis and storage, which is a key factor determining growth in crustaceans. Also, HMS is involved in nucleotide synthesis, and thus may also indicate a major growth potential of lobster on a fresh meat. The causes of these metabolic differences between fresh feed and formulated diets are currently unknown and still deserve investigation.

Diet composition is known to have a significant effect on the regulation of digestive α-amylase, mostly at the transcription level, in different invertebrate groups such as insects (Benkel & Hickey, 1987; Inomata et al., 1995), mollusks (Huvet et al., 2008; Huvet et al., 2012; Huang et al., 2016), and crustaceans (Rodríguez-Viera et al., 2016). It was found that lobsters fed with CHs sources such as corn, wheat and rice at 30% inclusion showed similar values of activity and expression of α-amylase, but when fed fresh fish (∼2% glycogen) both increased (Rodríguez-Viera et al., 2016). These results were taken as evidence of an effect of CH level on α-amylase gene expression. However, the CH level *per se* is not the cause of previously observed transcriptional variations, as this study covered a wide range of CH inclusion (6 to 35%) and no difference in transcript abundance was found. It is noteworthy, however, that a non-significant trend was observed, in which less α-amylase is expressed as starch increased (Fig. 3). Thus, the transcriptional regulation of α-amylase in lobster by CHs cannot be totally ruled out. However, there exists a general stimulatory effect of fresh fish meat on α-amylase expression and maybe other digestive enzymes. Therefore, the previous hypothesis on the role of high α-amylase activity in the gastric juice of fasted lobsters as sentinel for the CHs level of diet, and downstream regulation of α-amylase expression (Rodríguez-Viera et al., 2016), can be discarded. Current results are more in agreement with the genome simplification that the α-amylase gene in lobster has undergone through evolution (Rodríguez-Viera et al., 2016), probably as a result of a carnivorous feeding habits. It is now apparent that in addition to gene and intron losses in the lobster α-amylase gene (Rodríguez-Viera et al., 2016), transcriptional regulatory mechanisms have been simplified, being more responsive to unknown general signals from fresh food *vs*. test diets than to specific CHs level. However, while gene expression in the DG is similar in lobsters ingesting fresh fish (∼2% CH), 6% CHs, and 20% CHs, lobster fed fresh fish exhibited a higher activity in the gland in fasting animals. This finding suggests that there is a regulation of α-amylase activity at the secretion level. As DG enzyme activities cannot differentiate between the secreted enzymes and the enzymes that are stored as zymogens and activated as an artifact of tissue homogenization, we considered amylase activity in the gastric juice as a better indicator of enzyme secretion (in fasted lobsters, with no starch in gastric juice). Lobsters that usually ingested fresh fish had less α-amylase in the gastric juice during fasting (Fig. 1B, 30 h), while most enzymes remained stored in the DG (higher activity in the gland) (Fig. 3). This may be a reason for higher post-prandial response in α-amylase secretion in lobster fed fresh fish (Fig. 1B, 2–6 h). Also, note that lobsters fed on fresh fish were the only group decreasing gastric juice amylase during fasting (Fig. 1B, 30 h). Conversely, most test diets seemed to stimulate continuous enzyme secretion into the gastric juice under fasting conditions. This remarkable difference suggests that test diets may induce changes in the secretory activity of the DG, or produce overall structural changes in the organ that preclude the correct timing of α-amylase secretion after feeding.

## Concluding Remarks

In general, *P. argus* is able to digest and absorb CHs (e.g., wheat flour starch) efficiently. Most intense CHs digestion in the gastric juice of the lobster occurs during the first 6 h, which matches with the time course of the hyperglycemic response. The slow clearance of glucose from hemolymph in lobster ingesting 35% CHs indicates that such an inclusion level exceeds the capacity of lobster to utilize this nutrient. Some protein sparing effect of CHs exists in lobsters, but is limited by their intermediary metabolism to 20% CH. Regulatory mechanisms for digestive α-amylase activity in lobsters are complex and not completely understood, especially at the secretion level. Future studies are required to broaden this issue, as it may lie behind the inability of lobster to control the intensity and time-course of CHs digestion when fed formulated diets. Given that lobsters are not able to tightly regulate α-amylase expression in response to a wide range of dietary CH inclusion, high enzyme activity in gastric juice may have arisen as an adaptation to diets with few or moderate CH content. However, even when some anticipatory response (according to the usual CH content in diet) in gastric juice α-amylase activity was observed in this study, this may be not suited to control digestion of highly digestible CHs or high CH formulated diets, likely because of uncontrolled secretion of α-amylase into the foregut during fasting and its high activity in the conditions of the gastric juice (Perera et al., 2008a; Perera et al., 2008b; Rodríguez-Viera et al., 2016).

##  Supplemental Information

10.7717/peerj.3975/supp-1 Supplemental Information 1 Metabolites

10.7717/peerj.3975/supp-2 Supplemental Information 2 Metabolic enzymes

10.7717/peerj.3975/supp-3 Supplemental Information 3 Amylase expression and activity

## Additional Information and Declarations

### Data Availability

The following information was supplied regarding data availability: The raw data has been uploaded as  Supplementary Files.
